# Genetic association of E26 transformation specific sequence 1 polymorphisms with the susceptibility of primary biliary cholangitis in China

**DOI:** 10.1038/s41598-019-56181-9

**Published:** 2019-12-23

**Authors:** Huan Xu, Qian Niu, Zhenzhen Su, Fang Wang, Junlong Zhang, Bin Yang, Zhuochun Huang

**Affiliations:** 1 Department of Laboratory Medicine, West China Hospital, Sichuan University, Chengdu, China; 2 Department of Laboratory Medicine, The Second Affiliated Hospital, Guizhou Medical University, Guizhou, China

**Keywords:** Genetics, Immunology, Medical research

## Abstract

Primary biliary cholangitis (PBC) is a chronic and cholestatic liver disease characterized by an autoimmune-mediated destruction of intrahepatic bile ducts. E26 transformation specific sequence 1 (ETS-1) is a transcription factor regulating the expression of various immune-related genes. The aim of our study was to identify the associations between the gene polymorphisms of ETS-1 with the susceptibility and clinical characteristics of PBC in Chinese Han population. Three single nucleotide polymorphisms (rs4937333, rs11221332 and rs73013527) of ETS-1 were selected based on relevant studies. Genotyping was executed with polymerase chain reaction-high resolution melting (PCR-HRM) assay. SNP rs4937333 of ETS-1 was prominent correlation with the susceptibility of PBC (P = 0.007, OR = 1.44, 95%CI = 1.10–1.88). For rs4937333, PBC patients carrying the allele T assumed high-level TP (P = 0.020), and homozygous genotype TT assumed low-level RDW (P = 0.033). For rs11221332, PBC patients carrying the allele T assumed high-level TP and HDLC (P = 0.004, P = 0.015, respectively). For rs73013527, PBC patients carrying the allele T assumed low-level PLT (P = 0.002), and homozygous genotype TT assumed high-level RDW (P = 0.021). In conclusion, Gene polymorphisms of ETS-1 present relevant with the susceptibility of PBC, and affect the expression of TP, HDLC, PLT and RDW concentrations in patients with PBC.

## Introduction

Primary biliary cholangitis (PBC) is a chronic, progressive and cholestatic autoimmune liver disease characterized by the highly disease-specific autoantibody that is antimitochondrial antibody (AMA) and the histological destruction of small and medium-sized intrahepatic bile ducts that probably brings about fibrosis and sometimes even cirrhosis^1^. Incidence and prevalence of PBC are rising over time because of early diagnosis in the asymptomatic phase by the widespread use of automated blood testing, more effective serum auto-antibody detection methods, increased awareness and improved diagnostic abilities^2,3^. Although studies of the pathogenesis of PBC had made great progress in past decades, the precise mechanism of PBC is not extremely elucidated at present^4^. However, as with other autoimmune diseases, several pathogenetic factors have been considered to potentially trigger the onset of PBC, including infections^5^, environmental factors^6^ and genetic susceptibility^7^.

Genetic factors are considered to play a conspicuous role in disease onset of PBC^8,9^. Besides, scientists have expanded our knowledge of the genetic architecture of PBC through GWAS. To date, identified gene loci suggest that T lymphocyte differentiation plays a role in the development of PBC^10^. Pathological appearance of PBC is closely connected with peripheral blood cell subpopulations, especially T-regulatory (Treg) and T-helper 17 (Th17) lymphocytes, and both Treg and Th17 cells might play a crucial role in the pathogenesis and treatment of PBC^11^. E26 transformation specific sequence-1 (ETS-1) is a transcription factor that regulates the expression of various genes, including growth factors, chemokines and adhesion molecules, and it has been well demonstrated that ETS-1 plays a critical role in the proliferation and differentiation of immune cells such as T-cell differentiation into helper T lymphocytes^12^, and B cell into plasma cell^13^. Therefore, T lymphocyte differentiation is not only related to the pathogenesis of PBC, but also related to the regulatory mechanism of ETS-1. However, the association between pathogenesis of PBC and polymorphism of ETS-1 has not been analyzed.

Recently, the modern unit of genetic variation is now the single nucleotide polymorphism (SNP). SNPs are single base-pair changes in the DNA sequence and may fall within the coding regions of genes, non-coding regions of genes, or in intergenic regions^14^. In this aspect, association researches can determine whether a genetic variant is connected with PBC or not. To date, the correlation of PBC with many significant susceptibility SNPs, such as CTLA4^15^, TNF-α^16^, STAT4^17^ and PTPN22^18^, has been acknowledged over the past two decades.

For another, the variants of SNPs of ETS-1 were recognized as the significant correlation with autoimmune diseases, such as rs4937333 in systemic lupus erythematosus^19^, rs11221332 and rs73013527 in rheumatoid arthritis^20,21^. However, the association between susceptibility of PBC and genetic polymorphism of ETS-1 has not been reported. To better understand the role of ETS-1 in PBC, we analyzed the potential correlations between the three SNPs of ETS-1 and the susceptibility and the clinical characteristics of PBC in the southwest of China.

## Results

### Information of the study population

Our study included 151 PBC patients and 398 healthy controls and age and sex were well-matched between the two groups (P = 0.105 and P = 0.443, respectively). In the PBC patients, the mean level of TP concentration is 74.81 g/L; the mean of HDLC concentration is 1.50 mmol/L; the mean of ALP concentration is 235.11IU/L; the mean of PLT is 124.55 × 10^9^/L; the mean of RDW is 15.43%. The main demographic and clinical indicator information can be seen in Table 1.

**Table 1:** The main demographic and clinical indicator information of PBC patients and healthy controls.

Information	PBC(n = 151)	Control(n = 398)	P value
Age, mean ± SD (years)	55.69 ± 11.45	54.01 ± 8.82	0.105
Female (%)/ Male (%)	86.10/13.90	83.40/16.60	0.443
TP, mean ± SD (g/L)	74.81 ± 9.68		
HDLC, mean ± SD (mmol/L)	1.50 ± 0.71		
ALP, mean ± SD (IU/L)	235.11 ± 188.49		
PLT, mean ± SD (×10^9^/L)	124.55 ± 67.14		
RDW, mean ± SD (%)	15.43 ± 3.32		

Abbreviations: SD, standard deviation; TP, total protein; HDLC, high density lipoprotein cholesterol; ALP, alkaline phosphatase; PLT, platelet; RDW, red blood cell distribution width.

### Association of ETS-1 SNPs with the susceptibility of PBC

The three SNP genotypes corresponding to each individual were successfully and accurately obtained by using PCR-HRM method and direct sequencing of PCR products. Genotype frequencies from the HWE test showed no statistical difference in the target SNPs of the PBC patients or of the healthy controls (P > 0.05). The allele and genotype distributions of rs4937333 were significantly different between the two groups. The frequency of T allele of rs4937333 was significantly increased (P = 0.007, OR = 1.44, 95%CI = 1.10–1.88). Moreover, recessive model analysis showed that the frequency of TT genotype in PBC group was obviously increased (P = 0.003, OR = 1.98, 95%CI = 1.25–3.13), but the genotype frequency was no prominent difference in dominant model (P = 0.114, OR = 1.40, 95%CI = 0.92–2.11). For rs11221332 and rs73013527, no significant difference was found for either allele or genotype distributions between the PBC patients and healthy controls. Based on the Bonferroni correction, the p-value of 3 SNPs in the case-control study was less than 0.017 to be considered statistically significant, so the positive association between PBC and rs4937333 will remain. The allele and genotype distributions of ETS-1 can be seen in Table 2.

**Table 2:** Allele and genotype distributions of ETS-1 in PBC patients and healthy controls.

SNPs	Model	Type	PBC(n = 151)		Controls(n = 398)		OR (95%CI)	P value
			N	%	N	%		
Rs4937333	Allele	C	152	50.33	475	59.67	1.00	
		T	148	49.01	321	40.33	1.44(1.10–1.88)	**0.007**
	Dominant	CC	41	27.15	137	34.42	1.00	
		CT + TT	109	72.19	261	65.58	1.40(0.92–2.11)	0.114
	Recessive	CC + CT	111	74.00	338	84.92	1.00	
		TT	39	26.00	60	15.08	1.98(1.25–3.13)	**0.003**
Rs11221332	Allele	C	285	94.37	752	94.47	1.00	
		T	15	4.97	44	5.53	0.90(0.49–1.64)	0.730
	Dominant	CC	136	90.07	357	89.70	1.00	
		CT + TT	14	9.27	41	10.30	0.90(0.47–1.70)	0.737
	Recessive	CC + CT	149	99.33	395	99.25	1.00	
		TT	1	0.67	3	0.75	0.88(0.09–8.56)	0.915
Rs73013527	Allele	C	198	65.56	493	61.93	1.00	
		T	104	34.44	303	38.07	0.86(0.65–1.13)	0.266
	Dominant	CC	66	43.71	152	38.19	1.00	
		CT + TT	85	56.29	246	61.81	0.80(0.54–1.16)	0.238
	Recessive	CC + CT	132	87.42	341	85.68	1.00	
		TT	19	12.58	57	14.32	0.86(0.49–1.50)	0.598

Note: Data was presented as number and percentage for every group.

Significant p-values (<0.05) are highlighted in bold.

Abbreviations: OR, odds ratio; CI, confidence interval.

### Association of haplotype analysis of ETS-1 SNPs with the susceptibility of PBC

**Figure 1:**
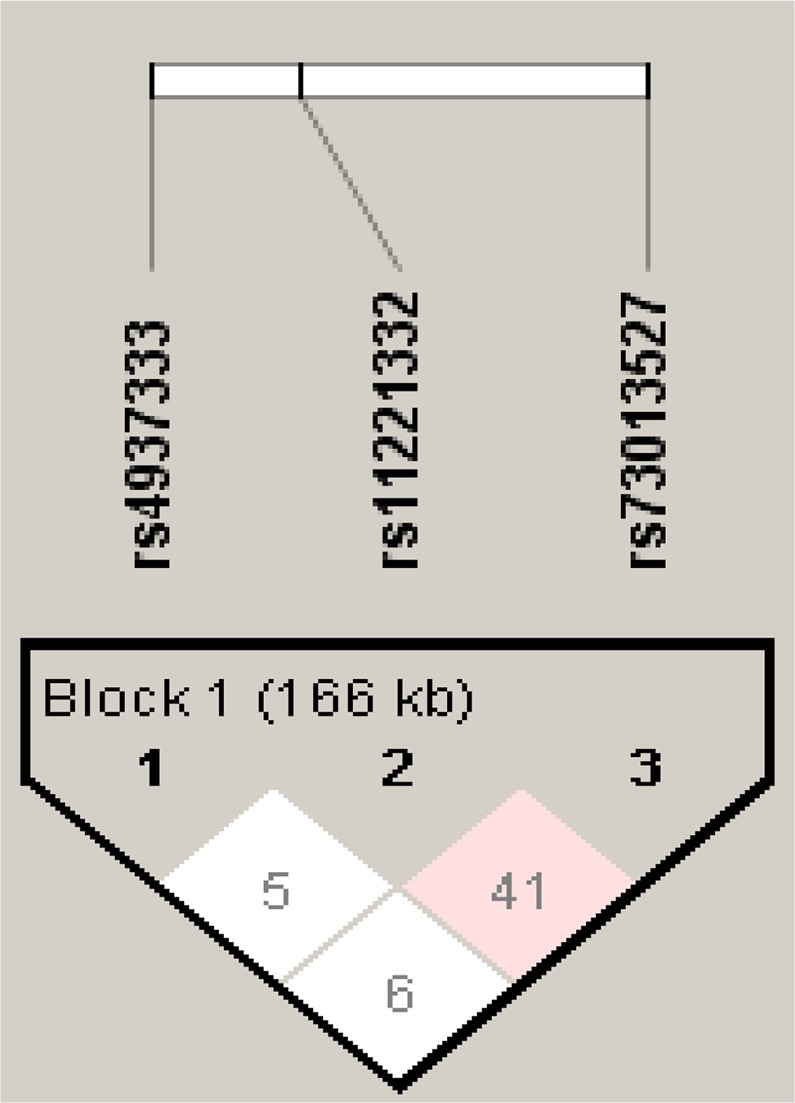
Linkage disequilibrium (LD) for three SNPs of ETS-1 in 549 individuals^36^. The LD plot shows the D’ value between each pair of SNPs. Between rs4937333 and rs11221332, D’ = 0.056 and r^2^ = 0.000; between rs4937333 and rs73013527, D’ = 0.062 and r^2^ = 0.002; between rs11221332 and rs73013527, D’ = 0.419 and r^2^ = 0.017. (Haploview 4.2 cited from https://sourceforge.net/projects/haploview/).

Our study selected three SNPs of ETS-1, namely rs4937333, rs11221332 and rs73013527, and obtained four common haplotypes based on frequency size (frequency >5%). The most common haplotype was the CCC, followed by TCC, CCT and TCT. Furthermore, their corresponding frequencies were 0.341, 0.269, 0.200 and 0.137, respectively. However, no association was found between ETS-1 haplotype and PBC susceptibility. In addition, haplotype analysis showed that the three loci of ETS-1 polymorphism were not in strong linkage disequilibrium. Haplotype analysis of ETS-1 can be seen in Table 3 and Fig. 1.

**Table 3:** Haplotype analysis of ETS-1 polymorphism in PBC patients and healthy controls.

Haplotype	All (freq.)	PBC (freq.)	Control (freq.)	OR (95%CI)	P value
CCC	374.42 (0.341)	97.00 (0.321)	277.00 (0.348)	1.000	
TCC	295.36 (0.269)	94.30 (0.312)	201.20 (0.253)	1.335 (0.954–1.870)	0.092
CCT	219.60 (0.200)	49.00 (0.162)	170.20 (0.214)	0.823 (0.556–1.219)	0.331
TCT	150.43 (0.137)	46.70 (0.155)	103.60 (0.130)	1.291 (0.852–1.954)	0.228

Note: Data was presented as number (percentage) for every group. Loci chosen for hap-analysis were in this order: rs4937333, rs11221332, rs73013527.

Abbreviations: OR, odds ratio; CI, confidence interval.

### Association of polymorphism of ETS-1 with the clinical characteristics of PBC

Our study showed that there was a significant correlation between three SNPs of ETS-1 and clinical characteristics of PBC. For rs4937333, genotype distribution showed no significant difference in the concentrations of TP, HDLC, ALP, PLT and RDW (P = 0.065, P = 0.123, P = 0.614, P = 0.910, P = 0.055, respectively), but the dominant model analysis showed that PBC patients carrying the allele T assumed high-level TP (P = 0.020), and the recessive model analysis showed that homozygous genotype TT assumed low-level RDW (P = 0.033). For rs11221332, the genotype distribution and dominant model analysis showed that PBC patients carrying the allele T assumed high-level TP and HDLC (TP: P = 0.012 and P = 0.004; HDLC: P = 0.030 and P = 0.015, respectively). For rs73013527, the genotype distribution and recessive model analysis showed that PBC patients carrying the allele T assumed low-level PLT (P = 0.010 and P = 0.002, respectively), and the recessive model analysis showed that homozygous genotype TT assumed high-level RDW (P = 0.021). After all p-values were corrected using Bonferroni for 15 tests (3 SNPs x 5 blood tests), we obtained one significant finding that rs73013527 was significantly correlated with PLT in the recessive model and showed low PLT. Association of polymorphism of ETS-1 with the clinical characteristics of PBC can be seen in Table 4.

**Table 4:** Association of ETS-1 polymorphism with the clinical characteristics of PBC.

SNPs	Model	Genotype	TP		HDLC		ALP		PLT		RDW	
			Median (P25, P75)	P value	Median (P25, P75)	P value	Median (P25, P75)	P value	Median (P25, P75)	P value	Median (P25, P75)	P value
rs4937333		CC	74.10(64.45,79.05)		1.22(0.66,1.86)		182.00(121.00,279.25)		110.00(61.50,182.50)		15.50(13.70,17.30)	
		CT	76.90(72.38,82.58)	0.065	1.59(1.20,1.83)	0.123	165.00(114.00,271.75)	0.614	112.00(80.25,151.25)	0.910	14.70(13.60,15.95)	0.055
		TT	77.20(73.20,82.00)		1.46(0.98,1.94)		193.00(136.00,312.00)		107.00(72.00,177.00)		14.00(13.10,16.00)	
	Dominant	CC	74.10(64.45,79.05)		1.22(0.66,1.86)		182.00(121.00,279.25)		110.00(61.50,182.50)		15.50(13.70,17.30)	
		CT + TT	76.90(72.70,82.10)	**0.020**	1.53(1.12,1.83)	0.053	171.00(115.00,290.00)	0.911	112.00(77.00,154.00)	0.813	14.40(13.20,15.95)	0.069
	Recessive	CC + CT	76.20(69.78,81.13)		1.51(1.03,1.83)		169.50(115.50,271.75)		111.00(76.00,161.00)		14.80(13.60,16.50)	
		TT	77.20(73.20,82.00)	0.333	1.46(0.98,1.94)	0.936	193.00(136.00,312.00)	0.382	107.00(72.00,177.00)	0.799	14.00(13.10,16.00)	**0.033**
rs11221332		CC	76.20(69.80,80.70)		1.47(0.98,1.83)		175.00(114.00,295.00)		110.00(72.25,155.00)		14.60(13.40,16.50)	
		CT	81.60(76.55,84.55)	**0.012**	1.77(1.63,1.87)	**0.030**	165.00(125.00,226.00)	0.519	191.00(90.50,221.50)	0.133	14.60(13.60,15.15)	0.393
		TT	86.30(86.30,86.30)		3.47(3.47,3.47)		344.00(344.00,344.00)		107.00(107.00,107.00)		13.00(13.00,13.00)	
	Dominant	CC	76.20(69.80,80.70)		1.47(0.98,1.83)		175.00(114.00,295.00)		110.00(72.25,155.00)		14.60(13.40,16.50)	
		CT + TT	81.80(77.88,85.40)	**0.004**	1.79(1.64,2.36)	**0.015**	171.00(128.00,259.00)	0.969	189.00(93.75,219.75)	0.056	14.60(13.48,14.98)	0.432
	Recessive	CC + CT	76.50(72.00,81.18)		1.51(1.01,1.83)		173.00(116.00,282.25)		111.00(73.00,162.00)		14.60(13.40,16.35)	
		TT	86.30(86.30,86.30)	0.143	3.47(3.47,3.47)	0.096	344.00(344.00,344.00)	0.264	107.00(107.00,107.00)	0.908	13.00(13.00,13.00)	0.200
rs73013527		CC	77.10(72.05,81.00)		1.50(1.04,1.97)		166.00(105.00,259.00)		117.50(84.00,172.00)		14.40(13.28,15.75)	
		CT	76.25(70.70,81.70)	0.845	1.60(1.00,1.88)	0.538	175.50(120.75,322.25)	0.570	111.50(75.25,179.50)	**0.010**	14.50(13.35,16.30)	0.070
		TT	75.60(72.00,82.00)		1.47(0.83,1.75)		185.00(121.00,283.00)		74.00(48.00,170.00)		16.10(14.10,17.50)	
	Dominant	CC	77.10(72.05,81.00)		1.50(1.04,1.97)		166.00(105.00,259.00)		117.50(84.00,172.00)		14.40(13.28,15.75)	
		CT + TT	76.20(71.80,81.80)	0.581	1.53(0.98,1.84)	0.838	180.00(121.00,304.00)	0.289	101.00(66.50,160.50)	0.195	14.60(13.40,16.55)	0.416
	Recessive	CC + CT	76.60(72.00,81.10)		1.53(1.03,1.88)		171.00(114.00,285.00)		116.00(81.50,176.25)		14.40(13.33,15.90)	
		TT	75.60(72.00,82.00)	0.726	1.47(0.83,1.75)	0.272	185.00(121.00,283.00)	0.697	74.00(48.00,170.00)	**0.002**	16.10(14.10,17.50)	**0.021**

Note: Significant p-values (<0.05) are highlighted in bold.

## Discussion

PBC is a complex chronic autoimmune liver disease characterized by immune-mediated biliary injury and cholestasis. ETS-1 is one of the most important transcription factors, which regulates a wide variety of cell growth and developmental processes. In this study, we have demonstrated gene polymorphisms of ETS-1 present relevant with the susceptibility of PBC and allele T of rs4937333 plays the role of risk factor in occurrence of PBC and ETS-1 gene loci assume significant differences about the level of TP, HDLC, PLT and RDW in patients with PBC in Chinese Han population.

The pathogenesis of PBC remains unclear. Recently, numerous genetic studies have led to the identification of risk loci for PBC^22^. In our study, the target is to find risk loci for PBC, but also to detect genetic polymorphisms that affect the progression of the disease. Indeed, we found a significant association between the polymorphism of ETS-1 and susceptibility of PBC. We identified the minor allele T of rs4937333 of ETS-1 was correlated with a high risk of susceptibility of PBC. In agreement with some autoimmune diseases such as systemic lupus erythematosus (SLE)^23,24^, rheumatoid arthritis (RA)^25^ and inflammatory bowel disease^26^, it was suggested that ETS-1was a susceptibility gene for PBC in southern Han Chinese. Therefore, the ETS-1 gene polymorphism seems to play a crucial role in the mutual susceptibility for autoimmune diseases.

Although our study showed that the SNP rs11221332 and rs73013527 of ETS-1 seemed not to be correlated with PBC susceptibility in Chinese Han population, the clinical features indicated significant associations, namely, patients carrying allele T of SNP rs11221332 presented high levels of TP and HDLC, and of SNP rs73013527 presented low levels of PLT and high levels of RDW. Scientists have yet fully elucidated the mechanism of increased serum levels of HDLC in patients with PBC. In another aspect, many scholars have found that RDW and RDW-to-platelet ratio (RPR) levels were associated with liver fibrosis and cirrhosis^27–29^ and might be used as a non-invasive indicator to predict the severity of liver histology^30^. It was interesting to note that RDW was correlated with the potential prognostic index of PBC^31^. However, the mechanism of RDW increase remains largely unknown in spite of several explanations that might indicate the elevated RDW levels. Firstly, biliary inflammation and injury in PBC are regulated by interleukin 12 (IL-12), interleukin 23 (IL-23) and interferon gamma (IFN-γ)^32^, so inflammatory cytokines might suppress the maturation of erythrocytes so that the newer and larger reticulocytes accelerate the entry into the peripheral blood circulation. Additionally, patients with PBC normally have hypersplenism due to portal hypertension, which might accelerate the destruction of RBCs. Thus, the immature RBCs that are larger than mature RBCs might be released from bone marrow into peripheral circulation leading to increased RDW. Finally, patients with liver disease often exhibit nutritional deficiencies, so their folic acid levels are usually lower compared with healthy controls, which could affect hematopoiesis and amplify the heterogeneity of RBC^30^. Unfortunately, haplotype analysis showed that no strong linkage disequilibrium was found.

ETS-1, one of the most important transcription factors among the ETS families, regulates the proliferation, senescence and death of multitudinous immune cells and plays a multifunctional role in autoimmune diseases. For example, the correlation between ETS-1 and miR-326 expression in CD19 + B cells is negative pathogenesis in Systemic lupus erythematosus patients^23^. Meanwhile, evidence continues to show that ETS-1 plays an important role in regulating the differentiation and development of immune cells such as T-cell differentiation into helper T lymphocytes^12^, B cell into plasma cells^13^, and the expression of cytokine and chemokine genes^33^ in a wide variety of different cells. Presented study demonstrated the correlation between PBC course and peripheral blood subpopulations, namely, the numbers of Treg cells reduced and Th17 cells increased could be responsible for the loss of immune tolerance, autoimmune process, inflammatory development, and liver fibrosis in PBC, and the progression of the disease might be the cause of Th17 cell activation and the release of IL-17^11^. Therefore, ETS-1 might play a crucial role in occurrence and development of PBC. Qiu^34^ *et al*.’s research supporting the hypothesis that IL21 signaling pathway and Tfh cells participate in the pathogenesis of PBC was a relatively large GWAS of PBC in the Han Chinese, but this study did not include the association between ETS-1 polymorphism and PBC susceptibility, so our study may be the first report that ETS-1 is a susceptibility site for PBC in the Han population.

There are several limitations of this study. Firstly, all PBC patients incorporated into the current study were southern Han Chinese so that the genetic background of many autoimmune conditions may be similar. Therefore, our results need to be confirmed in other populations in China and in European and East Asian populations. Secondly, although we clarified the correlation between minor allele T of rs4937333 of ETS-1 and a high risk of PBC susceptibility, we held the opinion that it might be only the tip of the iceberg. For further understanding the role of ETS-1 in the pathogenesis of PBC, more experiments are needed to determine its effect on differentiation of immune cells and the expression of cytokine and chemokine genes. Finally, all studies of ETS-1 have been conducted *in vitro*, but whether these observations represent performance *in vivo* remains an open question.

In conclusion, our study investigated the associations between polymorphism of ETS-1 and susceptibility and clinical characteristics of PBC. Our results demonstrated that the SNP rs4937333 of ETS-1 was associated with susceptibility and development of PBC and the SNPs rs11221332, rs73013527 of ETS-1 were correlated with TP, HDLC, PLT and RDW concentrations in patients with PBC with being exclusive of ALP. To our knowledge, this investigation was the first one executed in the population of China to assess the correlation between ETS-1 and PBC. Undoubtedly, our results laid a foundation in which we would like to continue to perform further scientific researches in the near future.

## Materials and Methods

### Participants

We screened 151 patients diagnosed with PBC from 5209 patients who had been tested for anti-mitochondrial antibody between September 2015 and December 2017 at the West China Hospital according to the diagnostic criteria of PBC in the American Association for the Study of Liver Diseases^1^. At the same time, 398 healthy controls were randomly selected among these people with normal physical examination and blood test and without underlying chronic diseases, epidemic infectious diseases, autoimmune diseases. Individuals participating in the case-control study signed informed consents, and the study conducted in accordance with the Declaration of Helsinki^35^ was approved by the Ethics Committee of West China Hospital.

### Clinical indicator information

The following information was gathered to analyze the association of ETS-1 polymorphisms with clinical characteristics of PBC. They’re age, gender, total protein (TP), high density lipoprotein cholesterol (HDLC), alkaline phosphatase (ALP), platelet (PLT) and red blood cell distribution width (RDW). Serological and hematological indicators of PBC were examined using the following methods: TP, HDLC and ALP were tested by Modular E170, Roche Diagnostics, Germany; PLT and RDW were tested by XN9000, Sysmex Diagnostics, Japan. All tests were conducted in accordance with manufacturers’ instruction.

### ETS-1 polymorphism genotyping

Considering that the genetic variation in rs4937333, rs11221332 and rs73013527 in ETS-1 might influence the immune response by regulating lymphocyte proliferation and differentiation process, the purpose of this study was to investigate the role of ETS-1 polymorphism in susceptibility to PBC. The genotyping took advantage of polymerase chain reaction-high resolution melting (PCR-HRM). The free circulating DNA in the peripheral blood was extracted by use of genomic DNA kit (Biotake Corporation, Beijing, China) and the concentration of DNA was performed by using Nanodrop 2000c spectrophotometer (Thermo Scientific, DE, USA). The whole genotyping process consisted of the following four procedures: pre-denaturation, amplification, high resolution melting and cooling on Light Cycler 480 (Roche Diagnostic, Germany). The results were analyzed with the corresponding Gene Scanning Software v1.2 (Roche Diagnostic, Germany).

### Haplotype analysis

The set of all SNPs alleles in the same chromosome region is called haplotype. Although the method of analyzing a single SNP may lead to meaningful discovery, haplotype analysis is a linkage disequilibrium (LD) association analysis based on SNP, which can locate complex disease genes and provide a promising method for detecting genetic variation of complex human diseases. Therefore, haplotype analysis was established on the basis of three SNPs of ETS-1 (rs4937333, rs11221332 and rs73013527) in this study. Table 3 and Fig. 1 were produced by the software Haploview 4.2 (https://sourceforge.net/projects/haploview/).

### Statistical analysis

The Hardy-Weinberg equilibrium (HWE) can evaluate whether genetic polymorphism meets requirements. Age and clinical data between case and control groups were compared with Student’s t test or Mann-Whitney U test appropriately. The differences of gender, allele and genotype between groups were analyzed with Pearson’s chi-square test or Fisher’s exact test. Estimating the association of SNPs with susceptibility of PBC was completed with the odds ratio (OR) and 95% confidence interval (CI). It was noteworthy that allele frequency distribution and genotype model of the two groups can use the following expressions: when A is the major allele and B is the minor allele, allele A versus allele B represents allele frequency distribution; AA versus AB + BB represents dominant model; AA + AB versus BB represents recessive model. Whether ETS-1 polymorphisms were in strong linkage disequilibrium (LD) was accomplished with the software HaploView 4.2 which may capture additional significant variants because it’s more sensitive than a single SNP analysis. Performing all the above statistical methods made use of the Statistical Package for the Social Sciences (SPSS, Chicago, USA) version 22.0. A two-sided P value < 0.05 was regarded as statistical significance.
